# Supplementary material for: Modified Technogenic Asphaltenes as Enhancers of the Thermal Conductivity of Paraffin
Source: Molecules. 2023 Jan 18;28(3):949. doi: 10.3390/molecules28030949 (PMC9920510; doi:10.3390/molecules28030949)
Supplement: Supplementary file 1 [file molecules-28-00949-s001.zip › molecules-2069555-supplementary.pdf]

Supporting information

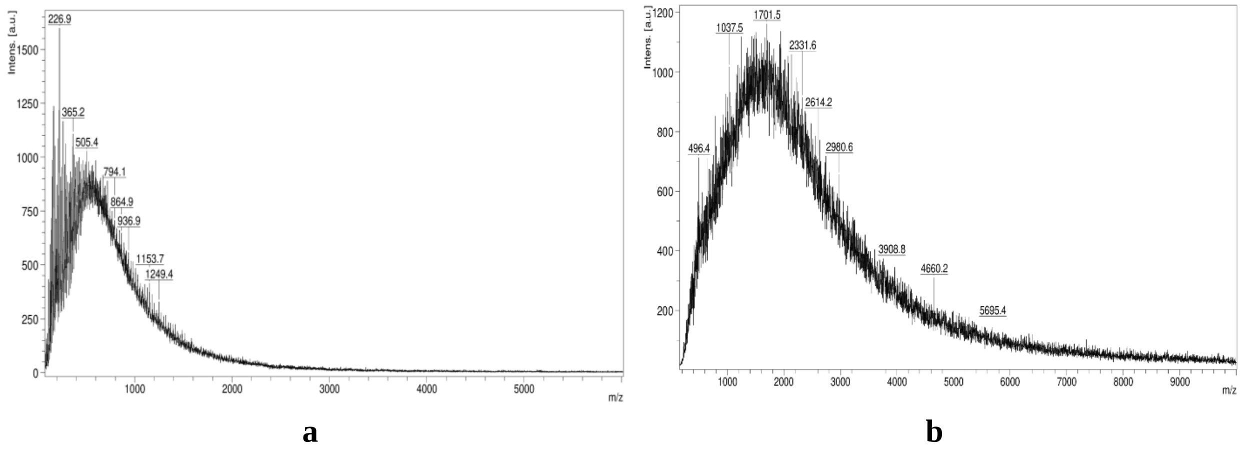

Figure S1. MALDI spectra of technogenic (a) and petroleum (b) asphaltenes.

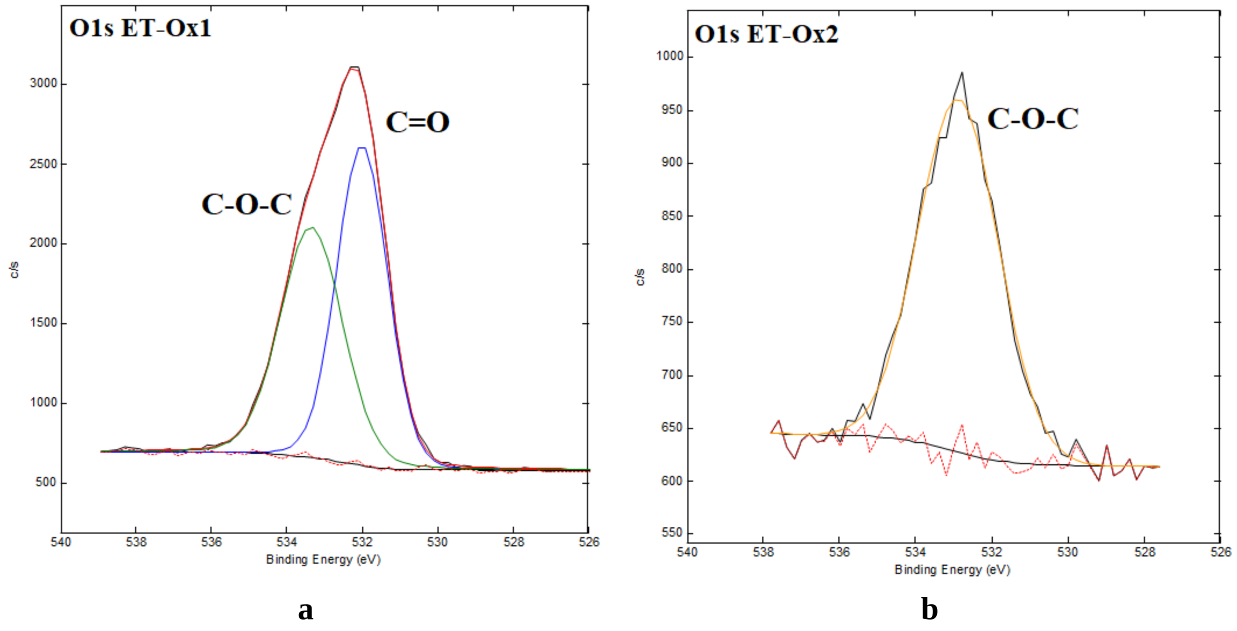

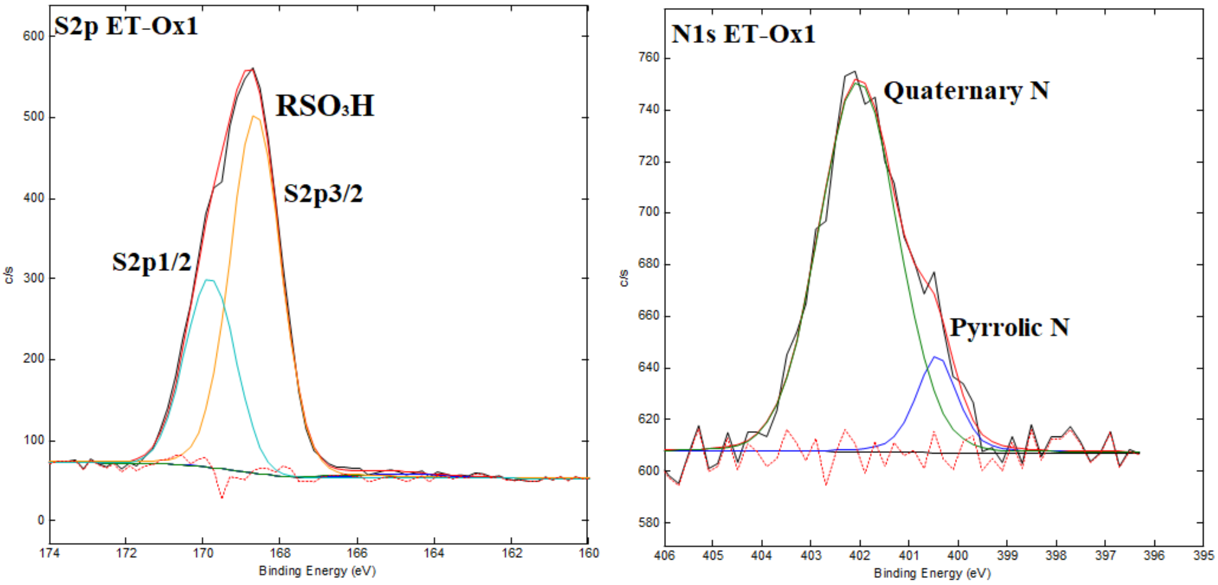

c d

Figure S2. XPS spectra of oxygen (a,b), sulfur (c) and nitrogen (d) in ET-Ox1 and ET-Ox2 products.
